# Functional Analysis of Dishevelled-3 Phosphorylation Identifies Distinct Mechanisms Driven by Casein Kinase 1ϵ and Frizzled5*

**DOI:** 10.1074/jbc.M114.590638

**Published:** 2014-07-03

**Authors:** Ondřej Bernatík, Kateřina Šedová, Carolin Schille, Ranjani Sri Ganji, Igor Červenka, Lukáš Trantírek, Alexandra Schambony, Zbyněk Zdráhal, Vítězslav Bryja

**Affiliations:** From the ‡ Institute of Experimental Biology, Faculty of Science, Masaryk University, Kotlarska 2, 61137 Brno, Czech Republic,; § Department of Cytokinetics, Institute of Biophysics, Academy of Sciences of the Czech Republic, Kralovopolska 135, 61265 Brno, Czech Republic,; ¶ National Centre for Biomolecular Research, Faculty of Science, Masaryk University, Kamenice 5, 62500 Brno, Czech Republic,; ‖ Research Group-Proteomics, Central European Institute of Technology (CEITEC), Masaryk University, Kamenice 5, 62500 Brno, Czech Republic,; ** Biology Department, Developmental Biology, Friedrich-Alexander University Erlangen-Nuremberg, Schloßplatz 4, 91054 Erlangen, Germany,; ‡‡ Structural Biology Program, Central European Institute of Technology (CEITEC), Masaryk University, Kamenice 5, 62500 Brno, Czech Republic, and; §§ Cellular Protein Chemistry, Utrecht University, Padualaan 8, 3584 CH Utrecht, The Netherlands

**Keywords:** Cell Signaling, Mass Spectrometry (MS), Phosphorylation, Post-translational Modification (PTM), Wnt Pathway, Casein Kinase 1ϵ, Dishevelled-3, Frizzled5

## Abstract

Dishevelled-3 (Dvl3), a key component of the Wnt signaling pathways, acts downstream of Frizzled (Fzd) receptors and gets heavily phosphorylated in response to pathway activation by Wnt ligands. Casein kinase 1ϵ (CK1ϵ) was identified as the major kinase responsible for Wnt-induced Dvl3 phosphorylation. Currently it is not clear which Dvl residues are phosphorylated and what is the consequence of individual phosphorylation events. In the present study we employed mass spectrometry to analyze in a comprehensive way the phosphorylation of human Dvl3 induced by CK1ϵ. Our analysis revealed >50 phosphorylation sites on Dvl3; only a minority of these sites was found dynamically induced after co-expression of CK1ϵ, and surprisingly, phosphorylation of one cluster of modified residues was down-regulated. Dynamically phosphorylated sites were analyzed functionally. Mutations within PDZ domain (S280A and S311A) reduced the ability of Dvl3 to activate TCF/LEF (T-cell factor/lymphoid enhancer factor)-driven transcription and induce secondar*y* axis in *Xenopus* embryos. In contrast, mutations of clustered Ser/Thr in the Dvl3 C terminus prevented ability of CK1ϵ to induce electrophoretic mobility shift of Dvl3 and its even subcellular localization. Surprisingly, mobility shift and subcellular localization changes induced by Fzd5, a Wnt receptor, were in all these mutants indistinguishable from wild type Dvl3. In summary, our data on the molecular level (i) support previous the assumption that CK1ϵ acts via phosphorylation of distinct residues as the activator as well as the shut-off signal of Wnt/β-catenin signaling and (ii) suggest that CK1ϵ acts on Dvl via different mechanism than Fzd5.

## Teaser

**Background:** Phosphorylation of Dishevelled (Dvl) by casein kinase 1ϵ (CK1ϵ) is a key event in Wnt signal transduction.

**Results:** Dvl3 residues phosphorylated by CK1ϵ were identified by proteomics and analyzed functionally.

**Conclusion:** Individual phosphorylation events control different aspects of Dvl biology.

**Significance:** CK1ϵ and Fzd5, a Wnt receptor, act on Dvl via distinct mechanism, suggesting that CK1ϵ is not directly downstream of Frizzled5.

## Introduction

The Wnt/β-catenin pathway is a conserved signaling machinery that coordinates cell proliferation, cell fate, and cell differentiation during development and during maintenance of homeostasis in the adult tissues (1, 2). Dishevelled (Dvl)^4^ with three isoforms in vertebrates (Dvl1, Dvl2, and Dvl3) is a key cytoplasmic protein required for signal transduction of the canonical Wnt pathway. Dvl is a modular protein composed of three domains, N-terminal DIX (Dishevelled, axin), central PDZ (postsynaptic density, discs large, zonula occludens), and DEP (Dvl, Egl-10, Pleckstrin). The linking regions between the domains do not seem to have well defined tertiary structure and might thus act as “hinge” regions, allowing Dvl to achieve various conformations. Although the reports about the function of individual domains are not always in full agreement, it is commonly acknowledged that DIX and PDZ domains are necessary for transduction of canonical Wnt signaling, whereas PDZ and DEP domains are necessary for other, so called non-canonical Wnt pathways (3–5). The part of Dvl located C-terminally from all three domains has no clear attributed function, although it was shown that it has inhibitory effects on canonical Wnt pathway (6).

Upon stimulation of cells by Wnt-3a, which is the best defined ligand of the Wnt/β-catenin pathway, Dvl electrophoretic mobility gets reduced due to multiple phosphorylations (7). Wnt-3a-induced activation of endogenous Dvl leads to a variant of Dvl with retarded electrophoretic mobility named phosphorylated and shifted (PS)-Dvl (8–10). It has been clearly demonstrated that both the activation of Dvl in the Wnt/β-catenin pathway (11–15) and Wnt-induced PS-Dvl formation are dependent on casein kinase 1 (CK1) δ/ϵ activity (8, 10). From the several protein kinases identified as Dvl binding partners, only CK1δ and CK1ϵ (for simplicity referred in further text as CK1ϵ) were shown to be required for Wnt-induced Dvl shift (11, 16) and at the same time capable of inducing PS-Dvl when overexpressed (13).

Serine, threonine, and tyrosine residues, which can be modified by phosphorylation, represent roughly 15% of Dvl amino acid residues. Despite the fact that many phosphorylation sites have been identified in Dvl (17–24), it is currently unknown which Dvl residues are dynamically phosphorylated by CK1ϵ. To overcome this caveat we have used a mass spectrometry (MS) based approach to identify in an unbiased way the phosphorylation sites of human hDvl3 in the absence and presence of overexpressed CK1ϵ. We have identified >50 phosphorylated Ser and Thr residues on Dvl3. The majority of these sites were found to be constitutively phosphorylated. Using criteria such as the site conservation, phosphorylation dynamics, and the position in Dvl3 we have selected several phosphorylation sites/clusters for mutation and subsequent functional analysis. Among these we have identified phosphorylation sites that (i) contribute to the electrophoretic mobility of Dvl3, (ii) control Dvl3-driven activation of the Wnt/β-catenin pathway, and (iii) are required for even subcellular localization of Dvl3.

## EXPERIMENTAL PROCEDURES

### Cell Culture, Transfection, and Treatments

HEK293t cells were propagated in DMEM, 10% FCS, 2 mm l-glutamine, 50 units/ml penicillin, 50 units/ml streptomycin. Cells (40,000–60,000 per well) were seeded in 24-well plates. The next day cells were transfected using polyethyleneimine (PEI) in a stoichiometry of 2.5 μl PEI per 1 μg of DNA. Cells were harvested for immunoblotting or immunocytofluorescence 24 h after transfection; for Western blot analysis of Frizzled (Fzd)-induced Dvl shift, samples were collected after 12 h. The following plasmids have been described previously: FLAG-Dvl3 WT (25), FLAG Dvl1 WT (26), FLAG-Dvl2 WT (27), CK1ϵ (28), dominant negative CK1ϵ P3 (15), and V5-Fzd5 (29). Cells were treated by 10 μm CK1 δ/ϵ inhibitor PF-670642 (Santa Cruz sc-204180). Mutagenesis was performed using the QuikChange XL kit following the manufacturer's instructions (Stratagene #200518). All mutations described in this study were verified by sequencing.

### Dual Luciferase Assay, Western Blotting, Ser(P)-643 Dvl3 Antibody Production, Immunoprecipitation, and Western Blot Quantification

For the luciferase reporter assay, cells were transfected with 0.1 μg of Super8X TopFlash construct and 0.1 μg of pRLTK luc (Renilla) luciferase construct per well in a 24-well plate 24 h after seeding. Cells were then transfected with the corresponding plasmids and processed 24 h after transfection. For the TopFlash assay, a Promega dual luciferase assay kit was used according to the manufacturer's instructions. Relative luciferase units of firefly luciferase were measured on a MLX luminometer (Dynex Technologies) and normalized to the Renilla luciferase signal. Immunoblotting and sample preparation was performed as previously described (10) The following antibodies were used: FLAG M2 (F1804, Sigma), CK1ϵ (sc-6471; Santa Cruz Biotechnology), V5 (R960–25, Invitrogen), FLAG (F7425, Sigma). Anti-hDvl3-Ser(P))-643 antibody was prepared by immunizing rabbits by HHSLAS(pS)LRSHH peptide. Immunization and production of antibody was performed on a service basis by Moravian Biotechnology. Western blot was quantified using ImageJ software. Briefly, area of the peaks for Ser(P)-643 antibody was divided by area of peaks for FLAG antibody, and statistical significance was confirmed by comparison of the control and PF-670642-treated conditions by Student's *t* test. Quantification was performed on *n* = 3, *p* > 0.05.

For MS/MS-based identification of phosphorylation, HEK293t cells were seeded on 15-cm dishes and transfected with corresponding plasmids 24 h after seeding. Immunoprecipitation protocol used was modified from Bryja *et al.* (31). In short, 2 ml of cold lysis buffer supplemented with protease inhibitors (Roche Applied Science, 11836145001), phosphatase inhibitors (Calbiochem, 524625), 0.1 mm DTT, and 10 mm *N*-ethylmaleimide (Sigma E3876) was used for lysis of one 15-cm dish. Lysate was collected after 20 min of lysis on 4 °C and was cleared by centrifugation at 16.1 relative centrifugal force (RCF) for 20 min. Three μg of antibody were used per sample. Samples were incubated with the antibody for 40 min, then 45 μl of G protein-Sepharose beads (GE Healthcare, 17–0618-05) equilibrated in the lysis buffer were added to each sample. Samples were incubated on the carousel overnight and washed 6 times with lysis buffer; 40 μl of 2× Laemmli buffer was added, and samples were boiled. The antibody used for immunoprecipitation was FLAG M2 (F1804; Sigma).

### Immunocytofluorescence

HEK293t cells were seeded (2 × 10^5^ cells/well) on gelatin-coated coverslips in 24-well plates. Cells were transfected the next day and 24 h later were fixed in fresh 4% paraformaldehyde, permeabilized with 0.05% Triton X-100, blocked with PBTA (3% BSA, 0.25% Triton, 0.01% NaN3) for 1 h, and incubated overnight with primary antibodies. The next day, coverslips were washed in PBS and incubated with secondary antibodies conjugated to Alexa Fluor 488 (Invitrogen A11001) or/and Alexa Fluor 594 (Invitrogen A11058), washed with PBS, stained with DAPI (1:5000), and mounted on microscopic slides. Cells were visualized using Olympus IX51 fluorescent microscope or Olympus Fluoview 500 confocal laser scanning microscope IX71. 100 positive cells per condition were analyzed and scored based on the pattern of intracellular Dvl3 distribution.

### Xenopus laevis Embryos

*X. laevis* embryos were generated and cultured according to general protocols and staged according to the normal table of Nieuwkoop and Faber (32). All procedures were performed according to the German animal use and care law (Tierschutzgesetz) and approved by the German state administration Bavaria (Regierung von Mittelfranken).

### Injection and Analysis of X. laevis Embryos and Tissue Explants

Capped RNA for microinjection was *in vitro* synthesized from the respective plasmids (pCS2-XDvl3 WT, pCS2-xDvl3 S279A, pCS2-xDvl3 S625A/S628A/S631A (C2 S-A), pCS2-CK1ϵ (11), pSP64 T3-xWnt8 (33)) using the mMessage mMachine kit (Ambion, Austin, TX). The following amounts of RNA were injected into the marginal zone of the ventral blastomeres of four-cell stage embryos: 0.5 pg of Wnt8, 500 pg of CK1ϵ, 500 pg of XDvl3 WT, 500 pg of XDvl3 S279A, 500 pg of XDvl3 C2 S-A. Embryos were cultivated at 17 °C until the desired stage, fixed in 1× MEMFA (3.7% formaldehyde, 100 mm MOPS, 2 mm EGTA, 1 mm MgSO_4_), and scored for the presence of secondary axes.

### Three-dimensional Predictions, Folding, Kinase Motives

Analysis and modeling of Dvl3 PDZ domain three-dimensional structure was performed using Chimera software (34). Folding predictions were generated by PONDR-FIT prediction software (35). Phosphorylation prediction was performed using GPS 2.1 program using predefined values for high, medium, and low stringency of the prediction (36).

### LC-MS/MS Analysis

Samples from immunoprecipitation were separated on a 8% SDS-PAGE, fixed with solution A (50% methanol, 10% acetic acid), then stained with Coomassie (0.1% Coomassie Brilliant blue (Sigma, BO149), 20% methanol, 10% acetic acid) for 2 h. Gel was destained using solution A. Corresponding one-dimensional bands were excised. After destaining, the proteins in gel pieces were incubated with 10 mm DTT at 56 °C for 45 min. After removal of DTT excess, samples were incubated with 55 mm iodoacetamide at room temperature in darkness for 30 min, then alkylation solution was removed, and gel pieces were hydrated for 45 min at 4 °C in digestion solution (5 ng/μl trypsin, sequencing grade (Promega, Fitchburg, WI) in 25 mm ammonium bicarbonate). The trypsin digestion was performed for 2 h at 40 °C on a Thermomixer (750 rpm; Eppendorf, Hamburg, Germany). Subsequently, the tryptic digests were cleaved by chymotrypsin (5 ng/μl, sequencing grade (Roche Applied Science) in 25 mm ammonium bicarbonate) for 2 h at 30 °C or 40 °C. Digested peptides were extracted from gels using 50% acetonitrile solution with 2.5% formic acid and concentrated in a SpeedVac concentrator (Eppendorf).

The aliquot (110) of concentrated sample was directly analyzed by LC-MS/MS for protein identification. The rest of the sample was used for phosphopeptide analysis. The sample was diluted with acidified acetonitrile solution (80% acetonitrile, 2% FA). Phosphopeptides were enriched using the Pierce Magnetic Titanium Dioxide Phosphopeptide Enrichment kit (Thermo Scientific, Waltham, MA) according to a slightly modified manufacturer protocol (samples were mixed with binding solution in a 1:2 ratio before loading and one additional washing step with binding solution after phosphopeptide capture was implemented). Eluates were concentrated under vacuum and then diluted in 10 μl of 0.1% FA solution before LC-MS/MS analysis.

Liquid chromatography-tandem mass spectrometry (LC-MS/MS) analysis was performed using reverse phase RSLCnano system (Dionex, Sunnyvale, CA) on-line-coupled with an HCT Ultra PTM Discovery System ion trap mass spectrometer equipped with electron transfer dissociation (ETD) source II (Bruker Daltonik, Bremen, Germany) or with an Orbitrap Elite hybrid spectrometer equipped by ETD, too (Thermo Fisher Scientific, Waltham, MA).

#### 

##### LC-MS/MS with HCT Ultra PTM Discovery System

Samples (10 μl) were injected in loading solution (0.1% FA) on a trapping column (100 μm × 30 mm) filled with 4-μm Jupiter Proteo sorbent (Phenomenex, Torrance, CA) where the analytes were desalted and concentrated. Subsequently, the analytes were eluted from the trapping column using an acetonitrile/water gradient (350 nl/min) onto a fused silica capillary column (100 μm × 180 mm) on which they were separated. The column was filled with 3.5-μm X-Bridge BEH 130 C18 sorbent (Waters, Milford, MA). The mobile phases A and B consisted of 0.1% formic acid in water and in acetonitrile, respectively. The gradient elution started at 4% of mobile phase B and increased to 30% B after 30 min and to 60% B in the next 10 min, then 90% B was reached in 1 min, and the system remained at this state for 5 min; finally the proportion of phase B was reduced to 4% B in 1 min. Equilibration of the precolumn and the column lasting 25 min was done before sample injection to sample loop. The analytical column outlet was directly connected to the nanoelectrospray ion source.

Mass spectrometer was operated in the positive ion mode with collision-induced dissociation followed, in the case of phosphopeptides, by data-dependent ETD fragmentation, triggered by detection of neutral loss of phosphoric acid in collision-induced dissociation spectrum. Nitrogen was used as nebulizing as well as drying gas. The pressure of nebulizing gas was 8 p.s.i. The temperature and flow rate of drying gas were set to 300 °C and 6 liters/min, respectively. The capillary voltage was 4.0 kV. Ion charge control controlling the filling of the ion trap was set to 200,000. The mass spectrometer scanned in *m*/*z* range of 300 −1500 for MS and 100–2500 for MS/MS operation. The data were processed with DataAnalysis 4.0 and BioTools 3.0 software (Bruker Daltonik).

##### LC-MS/MS with Orbitrap Elite Hybrid Spectrometer

Before LC separation, tryptic digests were on-line-concentrated and desalted using trapping column (100 μm × 30 mm) filled with 3.5-μm X-Bridge BEH 130 C18 sorbent (Waters). After washing of the trapping column with 0.1% FA, the peptides were eluted (300 nl/min) from the trapping column onto an Acclaim Pepmap RSLC C18 column (2 μm particles, 75 μm × 250 mm; Thermo Fisher Scientific) by the following gradient program (mobile phase A, 0.1% FA in water; mobile phase B, acetonitrile:methanol:2,2,2-trifluoroethanol (6:3:1; v/v/v) containing 0.1% FA); the gradient elution started at 2% mobile phase B and increased from 2% to 45% during the first 90 min (11% in the 30 min, 25% in the 60 min, and 45% in 90 min), then increased linearly to 95% of mobile phase B in the next 5 min and remained at this state for the next 15 min. Equilibration of the trapping column and the column was done before sample injection to sample loop. The analytical column outlet was directly connected to the Nanospray Flex Ion Source (Thermo Fisher Scientific).

MS data were acquired in a data-dependent strategy selecting up to top 10 precursors based on precursor abundance in the survey scan (350–1700 *m*/*z*). Mass spectrometer was operating in the positive ion mode with collision-induced dissociation followed, in case of neutral loss detection (32.7, 49.0, 65.3, and 98; with *m*/*z* tolerance of −1.5 and +0.5 Da), by ETD fragmentation with supplemental activation (energy 20, arbitrary units). The resolution of the survey scan was 120,000 (400 *m*/*z*) with a target value of 1 × 10^6^ ions, 1 microscan, and a maximum injection time of 200 ms. Low resolution collision-induced dissociation or ETD MS/MS spectra were acquired with a target value of 10,000 ions in rapid scan mode with the *m*/*z* range adjusted according to actual precursor mass and charge. MS/MS acquisition in the linear ion trap was carried out in parallel to the survey scan in the Orbitrap analyzer by using the preview mode. The maximum injection time for MS/MS was 50 ms. ETD reaction time was 100 ms (double-charged precursors, adjusted according to charge state). Dynamic exclusion was enabled for 45 s after one MS/MS spectra acquisition, and early expiration was disabled. The isolation window for MS/MS fragmentation was set to 2 *m*/*z*.

### Database Searching

The processed data were searched with MASCOT (Version 2.2 or higher, Matrix Science, Boston, MA) against NCBInr database (non-redundant, taxonomic restriction Mammalia) with settings corresponding to trypsin/chymotrypsin specificity (two miss-cleavages allowed) and optional modifications: oxidation (M), carbamidomethylation (C), phosphorylation (Ser, Thr, Tyr). Mass tolerances for peptides and MS/MS fragments were 0.5 Da in the case of HCT Ultra system (correction for 1 ^13^C atom) and 5 ppm and 0.5 Da, respectively, in case of Orbitrap system. The significance threshold was set to *p* < 0.05 and *p* < 0.01 for HCT Ultra and Orbitrap Elite generated data, respectively. All phosphorylation sites were manually confirmed in profile MS/MS spectra for generation of phosphorylation variants when necessary. The phosphoRS feature was used for phosphorylation localization in case of Orbitrap Elite data.

## RESULTS

### 

#### 

##### MS/MS-based Identification of Dvl3 Phosphorylation in the Presence and Absence of CK1ϵ

**FIGURE 1.**
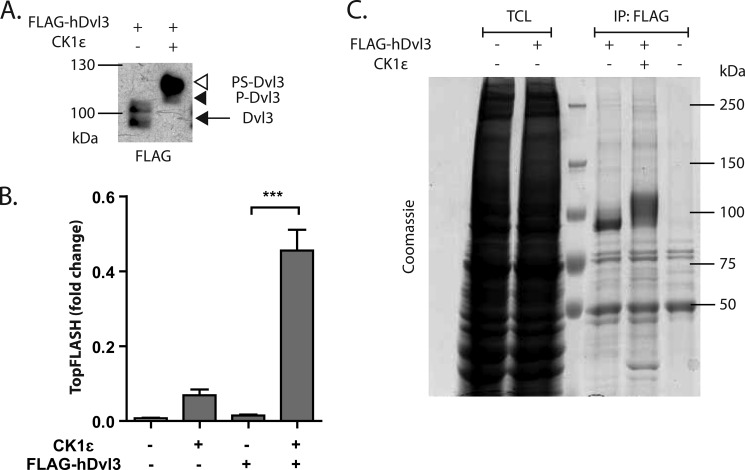
Co-expression of CK1ϵ with FLAG-Dvl3 retards electrophoretic migration and induces phosphorylation-dependent shift of Dvl (*PS-Dvl3*) (*A*) and induces TCF/LEF-dependent transcription as shown by TopFlash assay. Samples were analyzed by one-way analysis of variance followed by Tukey post tests. ***, *p* < 0.001, *n* = 3 (*B*). *C*, representative example of Coomassie Brilliant Blue R-stained gel used for MS/MS analysis. See the details “Experimental Procedures.”

CK1ϵ (together with the closely related CK1δ) is the only Dvl kinase that is both required and sufficient for the shift of Dvl electrophoretic mobility (Fig. 1*A*) and activation of the Wnt/β-catenin pathway (*e.g.* analyzed using the TopFlash reporter (37)) at the same time (Fig. 1*B*). Other kinases such as CK2, PAR1, RIPK4, protein kinase C (PKC), or Abl known to bind/activate Dvl upon overexpression do not lead to this phenotype (11, 16, 38–42). To identify Dvl3 phosphorylation sites controlled by CK1ϵ, we overexpressed FLAG-tagged human Dvl3 in HEK293t cells alone or together with CK1ϵ. Overexpressed Dvl3 was immunoprecipitated, separated on one-dimensional SDS-PAGE, and stained using Coomassie Blue (for typical gel see Fig. 1*C*). Bands were then excised from the gel, and phosphorylation was analyzed by LC-MS/MS.

**FIGURE 2.**
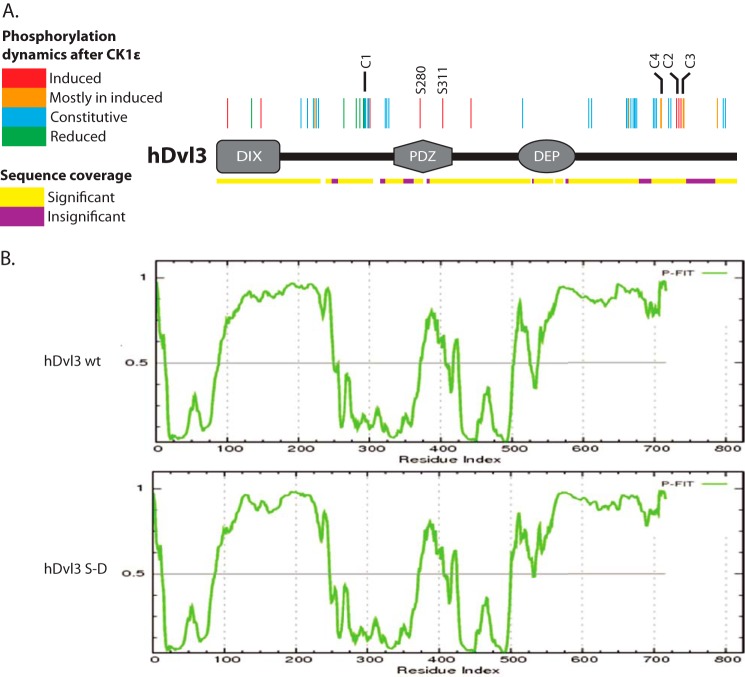
*A*, Dvl3 phosphorylation (summary of MS/MS data). Position and dynamics of detected phosphorylations is indicated by color coded lines: *green*, less frequent in CK1ϵ induced samples; *blue*, constitutive; *orange*, more often in CK1ϵ-induced samples; *red*, only in CK1ϵ-induced samples. The *yellow*/*violet lines* indicate hDvl3 protein coverage in the MS/MS analysis (*yellow*, significant identification; *violet*, insignificant identification). *C1*, *C2*, *C3*, *C4*, *Ser-280*, and *Ser-311* labels indicate the position of residues mutated in the study. *B*, secondary structure of Dvl3 as predicted by PONDR-FIT software (*upper panel*). Structured regions are indicated by values <0.5 and unstructured by values >0.5. Accuracy of the prediction is confirmed by the identification of DIX, PDZ, and DEP domains as regions with secondary structure. *In silico* mutation of all identified phosphorylated Ser/Thr sites in the basic region and C terminus for Asp did not lead to the massive changes in the secondary structure (*lower panel*).

Identification of phosphorylations in Dvl3/(Dvl3+CK1ϵ) samples was repeated in seven independent experiments. All significantly identified phosphorylation sites are summarized in Table 1 and Fig. 2*A*. Of >50 detected phosphorylation sites, only 9 were found uniquely in samples activated by CK1ϵ (Fig. 2*A*, in *red*). An additional seven serines were detected more frequently in the presence of CK1ϵ but were also occasionally seen in the control samples (Fig. 2*A*, in *orange*). We propose that phosphorylation of these 16 sites contributes to the Dvl3-associated phenotypes induced by CK1ϵ. Interestingly, phosphorylation of residues Ser-175, Ser-192, Ser-197, Ser-202, and Ser-203 in the region between DIX and the PDZ domain, which is rich in basic amino acids (the basic region), of Dvl3 was less abundant after CK1ϵ coexpression. These data suggest that some Dvl phosphorylation events may be inhibitory and mutually exclusive with others. Phosphorylation of the remaining 30 sites (Fig. 2*A*, indicated in *blue*) was not affected by CK1ϵ and seems to be constitutive in the used culture conditions. Most of the phosphorylation sites were found on residues conserved among Dvl isoforms and species (Dvl1, Dvl2, and Dvl3 of *Homo sapiens*, *Mus musculus*, *X. laevis*, and *Xenopus tropicalis* were considered for the analysis), but some were unique for Dvl3; this was the case of Ser-611, Ser-612, Ser-633 Ser-636, Ser-639, Ser-642, and Ser-643 in the His-rich region of the Dvl3 C terminus. For complete information about all identified phosphorylation sites, see Table 1.

**TABLE 1:** **Summary of hDvl3 phosphorylations**

Position*^a^*	Dvl/Dvl+CK1ϵ*^b^*	Presence*^c^*	Conservation*^d^*	CK1*^e^*	CK2*^e^*	PKC*^e^*	PLK1*^e^*	Peptide*^f^*	Note*^g^*
15	0/1	↑	**Y**				o	HLDGQE**T**PYLVKLPL	
48	1/0	↓	**Y**		X			RPSYKFFFK**S**MDDDFGVVK	
61	0/1	↑	Dvl2	X				FGVVKEEI**S**DDNAKLPCF	
116	1/1	↔	**Y**			X		GIGDSRPP**S**FHPHAGGGS	
125	6/6	↔	**Y**		X			HPHAGGG**S**QENLDND	
133	3/3	↔	y		X		X	QENLDND**T**ETDSLVS	
135	2/3	↖↗	y		X		X	NLDNDTE**T**DSLVSAQ	
137	3/3	↔	**Y**	X				DNDTETD**S**LVSAQRE	
140	4/4	↔	**Y**		X	X		TETDSLV**S**AQRERPR	
175	2/1	↓	**Y**		∼			RRREPGGYD**S**SSTLM	
192	7/5	↓	**Y**		X			ETTSFFD**S**DEDDSTS	
197	3/1	↓	Dvl2	X	X		X	FDSDEDD**S**TSRFSSS	
202	4/4	↓	**Y**		X		X	DDSTSRF**S**SSTEQSS	C1
203	7/4	↓	y		X	X		DDSTSRFS**S**STEQSS	C1
204	2/1	↔	**Y**		o	o	o	DDSTSRFSS**S**TEQSS	C1
205	3/6	↔	**Y**			∼	∼	TSRFSSS**T**EQSSASRL	C1
208	5/7	↔	Y			X		FSSSTEQ**S**SASRLMR	
209	5/7	↔	**Y**			X		FSSSTEQS**S**ASRLMR	
211	0/1	↑	**Y**	o		o		SSTEQSSA**S**RLMRRHK	
232	5/5	↔	Dvl2	X			X	KVSRIER**S**SSFSSIT	
234	6/5	↔	**Y**			X		KVSRIERSS**S**FSSIT	
237	6/4	↔	**Y**			o		ERSSSFS**S**ITDSTMS	
280	0/4	↑	**Y**					GGIYIG**S**IMKGGA	
311	0/4	↑	**Y**		X			EINFENM**S**NDDAVRV	
350	0/4	↑	n					CFTLPR**S**EPIRPID	
421	3/2	↔	n	X				AIVKAMA**S**PESGLEV	
512	2/3	↔	**Y**		X			SLHDHDG**S**SGASDQD	
516	4/5	↔	**Y**		o			DGSSGA**S**DQDTLA	
564	1/1	↔	**Y**				o	LGYSYGGG**S**ASSQHSEGS	
566	1/3	↔	n				X	SYGGGSA**S**SQHSEGS	
567	3/5	↖↗	**Y**			o		SYGGGSAS**S**QHSEGS	
570	6/4	↔	**Y**		X			GSASSQH**S**EGSRSSG	
573	5/6	↔	**Y**	X				SSQHSEG**S**RSSGSNR	
575	4/5	↔	**Y**		X			HSEGSR**S**SGSNRSGS	
576	4/5	↔	**Y**		X	X		HSEGSRS**S**GSNRSGS	
578	3/4	↔	**Y**			X		EGSRSSG**S**NRSGSDR	
601	3/4	↔	**Y**		X			GDSKSGG**S**GSESDHT	
603	4/3	↔	**Y**		X		X	GDSKSGGSG**S**ESDHT	
605	1/1	↔	**Y**				o	SGGSGSE**S**DHTTRSSLR	
611	1/3	↖↗	Dvl1				X	ESDHTTR**S**SLRGPRE	C4
612	1/3	↖↗	Dvl1			X		ESDHTTRS**S**LRGPRE	C4
622	2/6	↔	n			X		GPRERAP**S**ERSGPAA	
625	2/6	↔	n		o			RERAPSER**S**GPAASEHS	
633	0/1	↑	n			o		RSGPAASEH**S**HRSHHSLAS	C2
636	0/5	↑	n					ASEHSHR**S**HHSLASSLR	C2
639	0/6	↑	n				∼	EHSHRSHH**S**LASSLRSH	C3
642	1/3	↖↗	n			o		SHHSLA**S**SLRSHHTH	C3
643	1/5	↖↗	n			X		SHHSLAS**S**LRSHHTH	C3
689	2/5	↖↗	Dvl2					PPGRDLA**S**VPPELTA	
697	1/1	↔	**Y**			o		SVPPELTA**S**RQSFRMAMG	
700	1/1	↔	**Y**			X		ELTASRQ**S**FRMAMGN	

*^a^* Position of the amino acids.

*^b^* Number of identifications in Dvl/Dvl3+CK1ϵ; (found in *n* = experiments, *n* = 7).

*^c^* Presence of phosphorylation; ↑ only in induced; ↖↗ more in induced; ↔ constitutively phosphorylated; ↓ less in induced.

*^d^* **Y**, conserved in all isoforms (species considered: *M. musculus*, *H. sapiens*, *X. laevis*, *X. tropicalis*) as Ser/Thr/Tyr; y, conserved in all Dvl isoforms either as Ser/Thr/Tyr or polar amino acids (Asp, Glu, Gln, Asn); Dvl1/Dvl2, conserved also in this isoform; n, not conserved.

*^e^* X, high strindency prediction; o, medium stringeny prediction; ∼low stringency prediction.

*^f^* Phosphorylated peptide sequence^;^ Phosphorylation is in bold.

*^g^* C1/C2/C3/C4, mutated in clusters.

The MS/MS-based analysis demonstrated a complex pattern of Dvl3 phosphorylation and showed that many phosphorylation events take place independently of exogenous CK1ϵ. To classify individual phosphorylated motives, we searched the sequence of Dvl3 for substrate motives of known Dvl kinases, specifically CK1, CK2, PKC, and Polo-like kinase 1 (PLK1) using GPS 2.1 software (36). High stringency searches did not result in the prediction of kinases responsible for all phosphorylation sites; we thus present in Table 1 the results of searches with lower stringency. Surprisingly, this analysis showed that only two (Ser-61, Ser-211) of 16 phosphorylation events induced by CK1ϵ (indicated in *orange* or *red* in Fig. 2*A*) appeared in a canonical CK1 motif (see Table 1). Moreover, based on the software prediction, sequences containing Ser-280, Ser-350, and Ser-636 are not recognized by any of the considered Dvl kinases (CK1, CK2, PKC, and PLK1). This suggests that either CK1 recognizes atypical motives or alternatively that its phosphorylation sites are primed for subsequent phosphorylation by so far unidentified kinases. Our analysis does not provide definitive answer to that question.

Majority of the identified phosphorylated residues were found outside of the DIX, PDZ, and DEP domains (Fig. 2*A*). The only exceptions from this rule are infrequently detected Thr-15, Ser-48, and Ser-61 in the DIX and CK1ϵ-induced Ser-280 and Ser-311 in the PDZ domain. These three structured regions are linked by intrinsically unstructured regions (see PONDR-FIT prediction for hDvl3 in Fig. 2*B*) (35), which contained almost all phosphorylation sites (Table 1 and Fig. 2*A*). We have thus tested *in silico* whether or not the phosphorylations change the secondary structure and folding of the protein. Mimicking phosphorylation by changing phosphorylated Ser/Thr residues in the unstructured regions for aspartic acid (Dvl3 Ser-Asp) resulted in a slight increase of the score for the unstructured regions (Fig. 2*B*). However, the overall distribution of structured/unstructured regions was not massively affected, and it is unlikely that even robust Dvl3 phosphorylation at multiple sites affects the secondary structure of the protein. However, we cannot exclude minor changes or phosphorylation-induced formation of novel tertiary contacts.

##### Basal Dvl-induced TCF/LEF-driven Transcription Is Significantly Reduced by the Mutation of PDZ Domain Residues Ser-280 and Ser-311

To study the function of the repeatedly identified (detected more than once) phosphorylations, we decided to mutate the Ser/Thr residues, which were selected based on the following priority criteria: (i) unknown function, (ii) induction/repression by CK1ϵ, (iii) sequence conservation, and/or (iv) presence in the structured regions with the well defined function.

**FIGURE 3.**
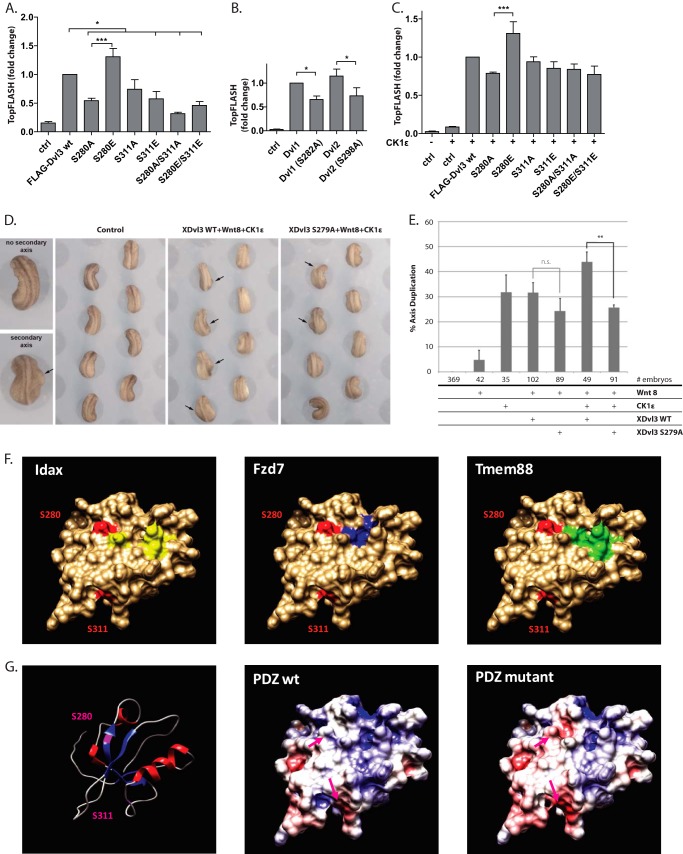
**Phospho-preventing mutations of Ser-280/Ser-311 in the PDZ domain blocks activation of the Wnt/β-catenin pathway.** *A–C*, HEK 293t cells were transfected with the indicated Dvl3 constructs, TopFlash reporter plasmid, plasmid encoding Renilla luciferase as an internal control, and CK1ϵ. *A*, mutations of Ser-280 and Ser-311 prevent efficient activation of Wnt/β-catenin by Dvl3. *B*, the function of Ser-280 is conserved in Dvl1 and Dvl2. Dvl1-S282A and Dvl2-S298A, corresponding to Dvl3 S280A, activated the Wnt/β-catenin pathway less efficiently than WT Dvl1 and Dvl2, respectively. *C*, in CK1ϵ-treated samples no significant differences between the WT Dvl3 and the mutant Dvl3 were detected. Samples were analyzed by one-way analysis of variance followed by Tukey post tests. *, *p* < 0.05; ***, *p* < 0.001; number of experiments ≥4. *D*, ventral overexpression of XDvl3 induced partial secondary body axes in *Xenopus* embryos. This phenotype reflects activation of the Wnt/β-catenin pathway *in vivo*. Images of one normal embryo and one embryo with a secondary axis are shown in larger magnification in addition to representative sets of embryos injected as indicated. The secondary body axes are indicated by *arrows. E*, the graph shows the average percentage of embryos that developed a secondary body axis from at least three independent experiments; *error bars* indicate the *standard error*, and *asterisks* indicate statistically significant deviation compared with overexpression of Dvl3-WT (*t* test, *p* > 0.99). *F*, interaction sites of Idax (in *yellow*), Fzd7 (in *blue*), and Tmem88 (in *green*) proteins determined for mouse Dvl1 PDZ domain (PDB ID 1MC7). Phylogenetically conserved Ser-280 and Ser-311 phosphorylation sites in human Dvl3 PDZ are shown in *red. G*, *left panel*, schematic representation of secondary structure of mouse Dvl1 PDZ domain. *Central panel*, modeled electrostatic surface potential of mDvl1 PDZ. *Right panel*, electrostatic surface potential of mDvl1 PDZ bearing phosphomimicking mutations at positions corresponding to Ser-280 and Ser-311 of human Dvl3 PDZ. Phosphorylation sites are highlighted in *magenta* (*magenta arrow*). Calculations of electrostatic surface potential were performed in UCSF Chimera software.

In the first step we functionally analyzed the phosphorylation of two individual serines: Ser-280 and Ser-311. These fully conserved residues are present in the central part of the PDZ domain, which is critically required for the function in the Wnt/β-catenin pathway. We mutated Ser-280 and Ser-311 of human Dvl3 to either alanine, to prevent phosphorylation, or to glutamic acid, to mimic constitutive phosphorylation and tested the ability of these mutants to activate the Wnt/β-catenin pathway by TopFlash assay. As we show in Fig. 3*A*, Wnt/β-catenin pathway induction by Dvl3 (S280A) was decreased, whereas induction by Dvl3 (S280E) was increased when compared with WT Dvl3. Ser-280 is conserved among all Dvl isoforms. To test the level of conservation of Ser-280 function, we have mutated the corresponding serines in Dvl1 (S282A) and Dvl2 (S298A). As shown in Fig. 3*B*, the mutations of serines corresponding to Ser-280 (hDvl3) to alanine in Dvl1 and Dvl2 also significantly reduced their ability to promote downstream signaling. These data suggest that phosphorylation of Ser-280 is required for the efficient Dvl activity in Wnt/β-catenin pathway across the whole Dvl family.

In contrast to Ser-280, both phospho-mimicking and phospho-preventing mutation of Ser-311 (S311A or S311E) resulted in a decreased activation of Wnt/β-catenin pathway. The function of Ser-311 phosphorylation, however, seems to be synergistic with Ser-280 as shown by the analysis double mutants. Mutation of Ser-280/Ser-311 to Ala or Glu almost completely abolished the activation of Wnt/β-catenin pathway by Dvl3 (Fig. 3*A*). Interestingly, when we co-expressed CK1ϵ (Fig. 3*C*), we did not observe any significant differences among individual mutants, although S280A and S280A/S311A mutants were slightly hypoactive, whereas S280E was hyperactive.

To confirm the impaired ability of Dvl3 S280A to signal in the Wnt/β-catenin pathway *in vivo*, we carried out axis duplication assays in *Xenopus* embryos. Ectopic activation of the Wnt/β-catenin pathway at the ventral marginal zone induced duplication of the dorsal body axis (43, 44). To avoid any cross-species effects, we have introduced the corresponding mutation in the *Xenopus* Dvl3 ortholog (XDvl3-S279A). We injected a sub-effective dose of *wnt-8* RNA combined with either *dvl3WT* or *dvl3S279A* RNA. The ability of XDvl3-S279A was slightly reduced compared with XDvl3WT, resulting in 25 and 32% axis duplication, respectively (Fig. 3, *D* and *E*). Additional co-expression of CK1ϵ combined with XDvl3 WT/Wnt-8 induced axis duplication in 44% of the embryos, which approximated a 1.5-fold increase of axis duplication compared with injection of *dvl3wt* + *wnt-8* RNA or single injection of *ck1*ϵ RNA. By contrast, CK1ϵ did not increase the percentage of axis duplication when co-expressed with XDvl3-S279A/Wnt-8 (Fig. 3, *D* and *E*), demonstrating that phosphorylation of XDvl3 at Ser-279 is required for full activation of Wnt/β-catenin signaling *in vivo*.

Data in Fig. 3, *A–E*, suggested an important role of Ser-280 and Ser-311 for the downstream canonical Wnt signaling. Specifically, phosphorylation at Ser-280 and subsequent electrostatic changes might be relevant because S280A decreases, whereas phospho-mimicking S280E increases the signaling efficiency of Dvl3. The observed functional phenotype might be explained by the fact that the Ser-280 directly borders with the binding area common to several proteins known to modulate Wnt signaling, namely Idax (45), Fzd7 (46), and TMEM88 (47) (Fig. 3*F*). Considering that the binding area for these proteins is predominantly positively charged, the introduction of a strong negative charge at its border is expected to produce a notable impact on binding affinity between the Dvl PDZ domain and the above-mentioned proteins (Fig. 3*G*).

##### Phosphorylation of Residues in the Dvl3 C Terminus Causes the Electrophoretic Mobility Changes Induced by CK1ϵ but Not by Fzd5

**FIGURE 4.**
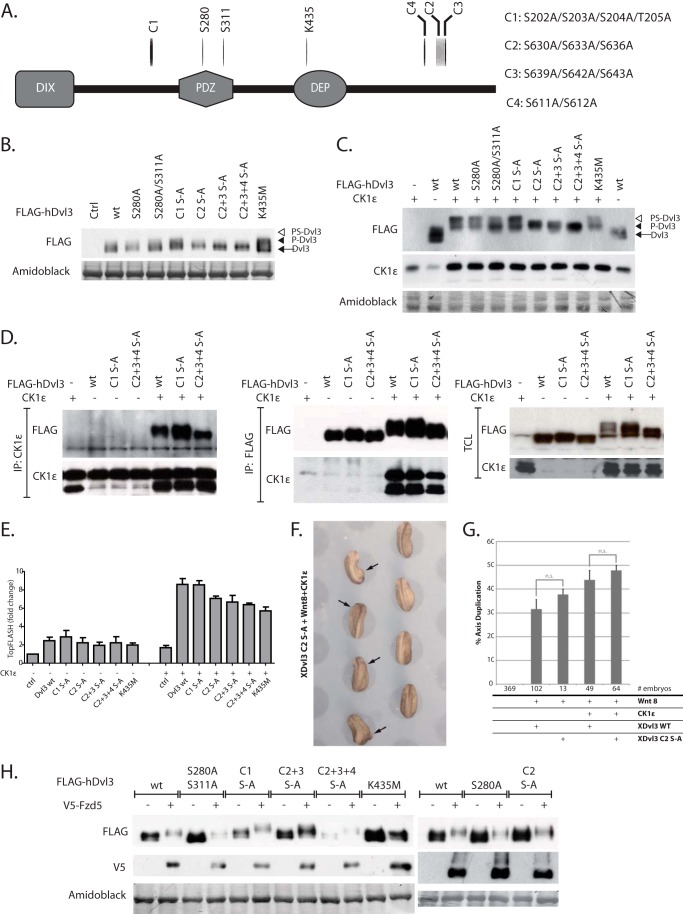
**Functional analysis of phosphorylation sites in clusters C1–C4.** *A*, representation of Dvl structure with the positions of mutated residues. *B* and *C*, HEK293t cells were transfected with the indicated plasmids, and the electrophoretic migration of Dvl3 in the absence (*B*) and presence (*C*) of CK1ϵ was analyzed by Western blotting with anti-FLAG antibody. Mutations in C2, C2+3, and C2+3+4 blocked PS-Dvl3 induction by CK1ϵ (*C*). The protein expression levels of individual mutants were comparable. *D*, the ability of individual Dvl3 mutants to interact with CK1ϵ was tested by immunoprecipitation (*IP*) of CK1ϵ and FLAG-Dvl3 and subsequent analysis by Western blotting (total cell lysate (TCL)). *E*, HEK293t cells were transfected with the indicated plasmids, and the ability to activate TCF/LEF-dependent transcription was assessed by TopFlash system. None of the C1, C2, C2+3, C2+3+4 mutants showed any statistically significant difference. Statistical differences were analyzed by one-way analysis of variance followed by Tukey post tests. Number of experiments *n* ≥ 3. *F* and *G*, XDvl3 S625A/S628A/S631A (XDvl3 C2 S-A) induced secondary body axes in *Xenopus* embryos at the same rate as XDvl3 WT. *F*, the image shows a representative set of embryos injected as indicated. *G*, the graph summarizes the average percentage of embryos that developed a secondary body axis from at least three independent experiments; *error bars* indicate the standard error. *n.s.*, not significant. *H*, HEK293t cells were transfected with the indicated plasmids, and the electrophoretic mobility of Dvl3 after V5-Fzd5 coexpression was analyzed by Western blotting. With the exception of Dvl3-K435M, electrophoretic mobility of all Dvl3 mutants was indistinguishable from wild type.

As the second step we mutated additional four clusters of Ser/Thr residues, which were chosen based on the criteria defined above. The mutated residues included the cluster of Ser-202/Ser-203/Ser-204/Thr-205 from the basic region (Cluster1 (*C1*)) and three C-terminally located clusters (Ser-630/Ser-633/Ser-636 (Cluster2 (*C2*)), Ser-639/Ser-642/Ser-643 (Cluster3 (*C3*)), and Ser-611/Ser-612 (Cluster4 (*C4*)) (Fig. 4*A*). The cluster C1 was selected because the level of phosphorylation of this region decreased after CK1ϵ co-expression, and these sites are conserved in all considered Dvl isoforms. The clusters C2, C3, and C4 are conserved only in Dvl3, but phosphorylation of these residues was strongly induced after CK1ϵ co-expression. The four clusters of residues were mutated to alanine.

Dvl can be detected on Western blots as two bands designated Dvl and P (phosphorylated)-Dvl. Co-expression of CK1ϵ promotes the formation of a novel even more slowly migrating form of Dvl3 named PS-Dvl (see Fig. 1*A*) that corresponds to the endogenous Dvl form induced by Wnts (8–10, 16). When we analyzed individual mutants, we found that they show rather comparable electrophoretic mobility (Fig. 4*B*). In contrast, co-expression of CK1ϵ led to the formation of PS-Dvl3 in all Dvl3 variants with the exception of Dvl3-C2, C2+C3, and C2+C3+C4 S-A mutants (Fig. 4*C*). The effects are not due to decreased ability of CK1ϵ to bind to Dvl3 because WT Dvl3 and its mutants C1 S-A and C2 + 3+4 S-A were able to bind to CK1ϵ with similar efficiency (Fig. 4*D*). The ability to activate TCF/LEF-driven transcription (TopFlash), which reflects the ability to activate Wnt/β-catenin pathway, was not significantly affected in any of the cluster mutants (Fig. 4*E*).

Again, we investigated the effect of the corresponding mutations in the *Xenopus* Dvl3 ortholog on Wnt/β-catenin signaling *in vivo* using the axis duplication assay. In contrast to the XDvl3-S279A mutant (Fig. 3, *D* and *E*), mutating serine residues 625, 628, and 631 (corresponding to cluster 2) in the C terminus of XDvl3 (XDvl3-C2 S-A) had no effect on its ability to induce secondary body axes in *Xenopus* embryos (Fig. 4*F*). We observed a slight increase in axis duplication when we co-expressed XDvl3-C2 S-A with sub-effective Wnt-8 or with Wnt-8 and CK1ϵ, but in both experiments the effects were statistically non-significant (Fig. 4*G*). Strikingly though, overexpression of XDvl3-C2 S-A with sub-effective Wnt-8 strongly affected the survival of the embryos, resulting in a very low number of embryos available for evaluation. This was not observed in embryos that additionally overexpressed CK1ϵ, indicating that CK1ϵ can amend the lethal effects of XDvl3-C2 S-A by an unknown mechanism. All together these observations suggest that residues in clusters C2, C3, and C4 are required for PS-Dvl formation but not for Dvl3 activity in the Wnt/β-catenin signaling.

It has been shown that not only CK1ϵ but also (Fzd) overexpression can lead to the electrophoretic mobility shift of Dvl. Wnt receptors Fzd are known to recruit Dvl to cytoplasmatic membrane and to trigger Dvl phosphorylation (5, 29). In the next step we thus tested the ability of Fzd5, selected for this study, to control electrophoretic migration of Dvl3 mutants. Co-expression of Fzd5 decreased the levels of Dvl3, which is likely due to the targeted degradation of Dvl3 recruited to the plasma membrane as described previously (48). Although Fzd5 was able to induce phosphorylation-dependent shift of Dvl3, the shift was never as prominent as PS-Dvl3 induced by CK1ϵ (Fig. 4*H*). Moreover, the intensity of Dvl3 phosphorylation was comparable in all Dvl3 mutants including Dvl3 C2+3+4 S-A (Fig. 4*H*). This opened the surprising possibility that CK1ϵ- and Fzd5-induced phosphorylation is qualitatively different. To test this possibility, we introduced K435M mutation in the DEP domain of hDvl3, which corresponds to fly *Dsh*^1^ mutant (Lys-417 in dDsh), which cannot interact with Frizzled (5). Surprisingly, the shift of Dvl3-K435M caused by CK1ϵ is comparable if not identical to that of WT Dvl3 (Fig. 4*C*), whereas Fzd5 is not able to trigger any detectable change in the mobility of Dvl3-K435M, in striking contrast to other mutants used in this study (Fig. 4*H*). This suggests that the phosphorylation events triggered by Fzd5 and CK1ϵ are not identical.

##### Phospho-preventing Mutations in Dvl3 C Terminus Interfere with CK1ϵ-induced but Not Fzd5-induced Changes in Dvl3 Subcellular Localization

**FIGURE 5.**
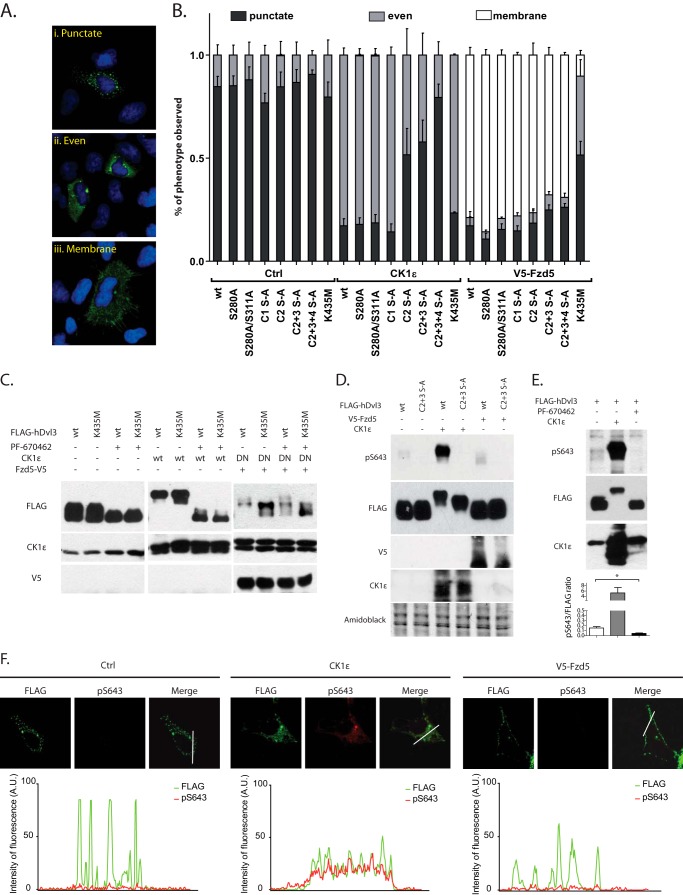
**Analysis of subcellular localization of individual Dvl pools.** *A*, representation of typical distribution patterns of Dvl3. (i), punctate; (ii), even, (iii), membrane. *B*, HEK293t cells were transfected with corresponding plasmids and fixed, and Dvl3 was stained with anti-FLAG antibody. Distribution pattern of Dvl3 was analyzed in at least 100 cells. CK1ϵ is unable to promote even localization of Dvl3 in C2, C3, and C4 S-A mutants, whereas V5-Fzd5 changes the intracellular distribution of Dvl3 to membranous in all tested constructs with the exception of Dvl3K435M, which served as a positive control. *C*, HEK293t cells were transfected with corresponding plasmids and treated as indicated with 10 μm CK1δ/ϵ inhibitor PF-670462. Inhibition of CK1δ/ϵ activity resulted in faster migration of FLAG-hDvl3 in control and CK1ϵ-induced conditions. In conditions with FLAG-hDvl3 coexpressed with V5-Fzd5 and dominant negative (*DN*) CK1ϵ-P3, acceleration of FLAG-hDvl3 electrophoretic mobility was observed only in mutant K435M. This finding confirms that CK1ϵ is and K435 is not dispensable for V5-Fzd5-induced phosphorylation of FLAG-hDvl3. *D*, phospho-Ser-643-Dvl3 was detected using anti-Ser(P)-643-Dvl3-specific antibody by Western blotting. Total Dvl3 was detected by anti-FLAG antibody. Anti-Ser(P)-643-Dvl3-specific antibody does not detect Dvl3 C2+C3 S-A mutant, but it strongly recognizes Dvl3 co-expressed with CK1ϵ. *E*, inhibition of endogenous CK1ϵ blocks signal detected by anti-Ser(P)-643-Dvl3 specific antibody. Decline in signal intensity was confirmed by Western blot quantification. CK1ϵ overexpression serves as a positive control. *F*, phospho-Ser-643-Dvl3 was detected by immunocytochemistry in HEK293t cells transfected with the indicated combination of plasmids. The signal of anti-Ser(P)-643-Dvl3 antibody (*red*) is negligible for Dvl3 expressed either alone or in combination with Fzd5 but strong for evenly distributed Dvl3 after CK1ϵ co-expression. Total Dvl3 detected by anti-FLAG antibody is shown in *green*. All confocal images were acquired using the same laser/detector settings and subsequently quantified using ImageJ software. Graphs show the overlap of fluorescence intensity peaks of individual channels along profiles indicated in the merged micrographs by a *white line. A.U.*, arbitrary units.

Dvl3 is usually found in dynamic multiprotein aggregates (49, 50) called Dvl dots or puncta. After coexpression of CK1ϵ, Dvl intracellular localization changes from punctate to even (for example, see Fig. 5*A*), a phenomenon that is associated with decreased polymerization of Dvl molecules (16). In contrast, high levels of Fzd receptor or Fzd co-expression (5, 29, 51) led to membrane recruitment of Dvl (Fig. 5*A*). In the next step we thus tested whether Dvl3 Ser/Thr-Ala mutants affect subcellular localization of Dvl3. As we show in Fig. 5*B* all Dvl3 mutants showed predominantly punctate localization pattern in HEK293t cells. Interestingly, after overexpression of CK1ϵ, the C1 mutant and K435M acquired even localization similarly to WT Dvl3, whereas C2, C2+3, and C2+3+4 S-A mutants showed an additive deficit in their ability to achieve even subcellular distribution. Localization of the C2+C3+C4 S-A mutant in the presence and absence of overexpressed CK1ϵ was indistinguishable. Mutations within PDZ domain showed no detectable effect on localization pattern of Dvl3 following CK1ϵ overexpression.

In contrast to CK1ϵ, none of the tested Dvl3 S-A mutants showed a deficit in the Fzd5-mediated recruitment of Dvl3 to the plasma membrane (Fig. 5*B*). Dvl3 (K435M), which is analogous to *Drosophila* dDsh mutant *Dsh*^1^ (5) and is expected to be deficient in membrane recruitment by Fzd, however, clearly failed to be recruited to plasma membrane by Fzd5 co-expression (Fig. 5*B*). This suggests that residues phosphorylated in response to CK1ϵ and mutated in this study are not required for Fzd5-induced membrane localization of Dvl3.

In combination with the data in Fig. 4, we propose that membrane recruitment of Dvl3 is required for phosphorylation events induced by Fzd5 but not by CK1ϵ. To test this prediction biochemically, we analyzed whether Fzd5-induced Dvl3 shift depends on the activity of CK1. As we show in Fig. 5*C*, CK1 activity is required for the retarded electrophoretic mobility of wild type as well as K435M-Dvl3 in both CK1ϵ-induced and basal conditions. However, Fzd5 overexpression was capable of inducing a phosphorylation-dependent mobility shift of wild type but not K435M-Dvl3 even in the presence of dominant negative CK1ϵ (Fig. 5*C*, *last four lanes*). Moreover, we were unable to block this shift by additional pharmacological inhibition of CK1ϵ, which together strongly suggest that Fzd5-induced events on Dvl3 are independent of CK1ϵ but require intact DEP domain and membrane recruitment.

##### Phospho-Ser-643 Antibody Recognizes CK1ϵ-phosphorylated and Evenly Distributed Dvl3

To get better insight into the subcellular localization of individual phospho pools of Dvl3, we raised a phospho-specific antibody recognizing phosphorylated Ser-643. We selected phosphoserine 643 as a candidate epitope because it belongs to the cluster of residues phosphorylated after CK1ϵ coexpression, and it is the last phosphorylated residue in the cluster of Ser(P)-633, Ser(P)-636, Ser(P)-639, Ser(P)-642, and Ser(P)-643. As we demonstrate on Western blotting, anti-Ser(P)-643-Dvl3 antibody strongly detected Dvl3 co-expressed with CK1ϵ, whereas it almost did not recognize FLAG-Dvl3 expressed alone or in combination with Fzd5 (Fig. 5*D*). Anti-Ser(P)-643-Dvl3 antibody failed to detect Dvl3 (C2+3 S-A) mutant where Ser-643 is mutated to Ala-643, which demonstrates that it is specific. Phosphorylation of Dvl3 at Ser-643 is not only promoted by exogenous CK1ϵ, but it also depends on the endogenous CK1ϵ activity as demonstrated by the complete lack of the Ser(P)-643 signal in cells treated with the CK1 inhibitor PF670462 (Fig. 5*E*).

Next we analyzed subcellular distribution of Ser(P)-643-Dvl3 by immunocytochemistry to identify the pool of Dvl3 specifically modified by phosphorylation in the C terminus. The staining showed a weak signal that was dramatically boosted by CK1ϵ overexpression, causing even localization of Dvl3 (Fig. 5*F*). Importantly, Fzd5 overexpression leading to Dvl3 membrane recruitment did not increase the Ser(P)-643-Dvl3 signal. This suggests that membranous, Fzd5-recruited Dvl3 is not modified by CK1ϵ at Ser-643. This observation correlates well with the analysis of Dvl3 electrophoretic shift (Figs. 4*C* and 5*C*) and demonstrates that phosphorylation of C2/C3/C4 residues is part of the mechanism that promotes even localization of Dvl3 and is distinct from the signaling events induced by Fzd5.

## DISCUSSION

In this study we performed unbiased proteomic identification of Dvl3 phosphorylation pattern induced by CK1ϵ. Previous studies in various experimental systems identified several phosphorylation sites spread throughout the structure of Dvl protein (18, 20–22). Combination of all data clearly demonstrates that phosphorylation of Dvl protein is extensive and the number of phosphorylated sites exceeds 50. However, it is likely that the number of phosphorylated residues on a single Dvl molecule is lower because MS/MS analysis cannot distinguish individual differentially modified Dvl pools. This assumption is supported by our observation that despite a general increase in the number of phosphorylated residues, some phosphorylation sites in the basic region of Dvl3 disappeared after CK1ϵ coexpression.

To our surprise, the majority of the detected phosphorylation events were not promoted by CK1ϵ. This suggests that Dvl3 gets heavily phosphorylated before Wnt-induced CK1ϵ-mediated activation. This is in good agreement with our previous work where we show that CK2 mediates such a basal state of Dvl phosphorylation, which is then required for dynamic phosphorylation by CK1ϵ and for further downstream signaling in the Wnt/β-catenin pathway (16). Several kinases including PKC, CK2, PLK1, RIPK4, and Abl have been implicated previously in Dvl binding and phosphorylation (11, 19, 39–42), and our *in silico* analysis of phosphorylation sites support possible involvement of these kinases in the basal state Dvl phosphorylation.

Our findings support our earlier assumption that CK1ϵ has a dual function in Dvl biology (16). It seems that CK1ϵ acts as (i) the activator of downstream Wnt/β-catenin signaling via phosphorylation of distinct Ser/Thr residues (phosphorylation of Ser-280/Ser-311 in the PDZ domain) as well as (ii) the inactivating kinase affecting Dvl polymerization via phosphorylation of the residues (C2+C3+C4) in the C terminus of Dvl3. The role of the Dvl C terminus in the Wnt/β-catenin signaling was established by our previous work, where we proposed that it acts as the CK1ϵ-controlled negative regulator (6, 16). Here we identified specific residues phosphorylated by CK1ϵ in the C terminus of Dvl3 (clusters C2, C3, and C4). We demonstrate that these residues control PS-Dvl formation and are critical for CK1ϵ-induced changes in Dvl3 subcellular localization. Although the clusters C2, C3, and C4 are conserved only in Dvl3, multiple Ser/Thr residues are present also in the C-terminal region of Dvl1 and Dvl2. This opens the possibility that the function of the phosphorylated C terminus is conserved despite the differences in the primary sequence. This possibility is supported by recent findings by González-Sancho *et al.* (17), which demonstrated that formation of PS-Dvl2 and its even localization depends on other residues of the hDvl2 C terminus. The residues are conserved in Dvl3 (Ser-578 and Ser-581 in hDvl3) but were found constitutively phosphorylated in our study.

Our data support the line of recent evidence that the Dvl C terminus and the phosphorylation therein control the activity of Dvl in Wnt/β-catenin pathway. It was reported that Dvl C terminus binds several components of the non-canonical Wnt pathway such as NFAT (nuclear factor of activated T cells) (52) or Ror2 (6). This together with other reports (53) suggests that the Dvl C terminus can critically contribute to several branches of non-canonical Wnt signaling. In line with these observations, it was recently shown that the phosphorylation of the residues in the C terminus of Dvl2 necessary for PS-Dvl2 formation is required for Dvl2-dependent neurite outgrowth in TC-32 cells, a process not mediated by Wnt/β-catenin pathway (17). It is not well understood how Dvl C terminus exerts its function; however, a recent report proposes that the C terminus can intramolecularly interact with the PDZ domain (30). Phosphorylation in the C terminus can then affect the intramolecular conformation similarly to the regulatory mechanism well described among protein kinases. This possibility is supported by our earlier analysis of the interaction of Ror2 and Dvl3, which showed that Ror2 could efficiently recognize only PS-Dvl3 or the Dvl3 C terminus as such (6).

The most comprehensive study to date, which attempted to identify Dvl phosphorylation, was performed in the *Drosophila* model and used activation of Dvl by Frizzled overexpression (18). This study concluded that Ser/Thr phosphorylation of Dvl has no role either in PCP or canonical Wnt signaling in the fly. In comparison to that study we tested phosphorylation of Dvl3 by overexpression of the most relevant Dvl kinase, CK1ϵ. To our surprise, when we mutated the most dynamically phosphorylated Dvl3 residues in the C terminus, we found that they are deficient only in CK1ϵ-induced but not in Fzd5-induced mobility shift or subcellular localization changes. These distinct molecular features of CK1ϵ- and Fzd5-induced events were further confirmed by implementing anti-Ser(P)-643-Dvl3 phospho-specific antibody, which recognized only Dvl3 modified by CK1ϵ but not by Fzd5. These data suggest that CK1ϵ is not directly downstream of Fzd receptor but rather represents an independent, possibly parallel, molecular mechanism of activation/de-activation of Dvl.

In summary, our findings shed more light onto the complex molecular events on the level of Dvl3 phosphorylations triggered by CK1ϵ. On the single amino acid level we show that CK1ϵ-mediated phosphorylation of Ser-280 and Ser-311 in the PDZ domain is one of the steps in the chain of events leading to Wnt/β-catenin downstream activation. On the contrary, phosphorylations in the C terminus contribute to the change of Dvl3 electrophoretic mobility and mediate negative effects of CK1ϵ on Dvl polymerization, which were postulated to be part of the negative feedback loop.

## Footnotes

4 The abbreviations used are:

Dvl: Dishevelled
PDZ: postsynaptic density, discs large, zonula occludens
DEP: Dvl, Egl-10, pleckstrin
DIX: Dishevelled, axin
PS: phosphorylated and shifted
hDvl3: human Dvl3
ETD: electron-transfer dissociation
FA: formic acid
PLK1: Polo-like kinase 1
Fzd: Frizzled
TCF/LEF: T-cell factor/lymphoid enhancer factor
RCF: relative centrifugal force
TCL: total cell lysate.
